# A Critical Overview of the Validity of the Current Concept of Bipolar Disorder

**DOI:** 10.3390/jpm15120624

**Published:** 2025-12-12

**Authors:** Diego J. Martino, Alejandro G. Szmulewicz, Boris Birmaher

**Affiliations:** 1 National Council of Scientific and Technical Research (CONICET), Buenos Aires C1425FQB, Argentina; 2 Institute of Cognitive and Translational Neuroscience (INCyT), INECO Foundation, Favaloro University, Buenos Aires 1056, Argentina; 3 Department of Epidemiology, Harvard TH Chan School of Public Health, Boston, MA 02115, USA; 4 Western Psychiatric Institute and Clinic, University of Pittsburgh School of Medicine, Pittsburgh, PA 15213, USA; birmaherb@upmc.edu

**Keywords:** bipolar disorder, manic-depressive, diagnosis, mania, hypomania, melancholia, depression

## Abstract

**Objectives**: The main aim was to evaluate the origin and empirical support of the current diagnostic criteria for (hypo)manic and depressive episodes in BD focusing on their nosological (i.e., is it a real entity?) and diagnostic validity (i.e., how well do the criteria for the category portray the entity?). **Methods**: A narrative review of relevant textbooks/reports and articles published in peer-reviewed English-language journals (from the online databases PubMed and PsycInfo), covering the period 1900–2024 and using the terms “validity” OR “diagnosis” AND “manic-depressive”; “mania”; “hypomania”; “depression”; and “melancholia” was performed. **Results**: Mania appears to be a valid construct in nosological terms, although its validity in the diagnostic domain requires further research. There are scant and controversial empirical data on the nosological validity of separating hypomania from mania as different episodes. The current concept of bipolar depression combines different forms of episodes (melancholic and non-melancholic, with or without psychosis, recurrent or not) without conclusive evidence that all of them are necessarily part of the illness (i.e., limited nosological validity). **Conclusions**: The validity of the current definition of BD is limited and should be the focus of future research. A valid definition of BD would improve our ability to understand the pathophysiological basis of the illness and contribute to more tailored therapeutic approaches.

## 1. Introduction

Although several neuroimaging and other biomarkers have been associated with bipolar disorder (BD), the results are inconsistent and overlap with those described in other psychiatric disorders [1,2]. Findings of the genome-wide association studies of BD are also inconsistent and correlated with other mood and psychotic disorders [3,4]. Faced with these facts, it has been proposed to abandon the traditional diagnostic categories in favor of a dimensional view of psychopathology, or to develop other research frameworks such as the Research Domain Criteria [5,6,7]. An alternative approach advocates, rather than abandoning the extant diagnostic categories, validating them based on data derived from clinical research as an essential step to advance our knowledge on BD. In a recent review, a group of well-known researchers argued that “If the clinical phenotype for bipolar illness is wrong, imprecise or heterogeneous, genetic studies will fail, biological marker studies will be inconsistent, and treatment studies will be ineffective” [8]. The rationale for this proposal lies in the fact that the current categories derive with minimal changes from the Research Diagnostic Criteria (RDC) [9] and the DSM-III (published in 1980). These categories emerged from a consensus of experts focused on improving the reliability of diagnoses, an urgent need at the end of the 1970s, although somewhat relegating its validity [8,10].

To address this issue, the first aim of this narrative review was to evaluate the origin and empirical support of the current diagnostic criteria for (hypo)manic and depressive episodes in BD (i.e., those episodes included in official nosologies such as the DSM-5) focusing on their validity. To that end, articles published in peer-reviewed, English-language journals between 1900 and 2020 were retrieved from the online databases PubMed and PsycInfo using the search terms “validity” OR “diagnosis” AND “bipolar”; “manic-depressive”; “mania”; “hypomania”; “melancholia”; “depression”. The reference lists of the studies identified for inclusion were also reviewed for further relevant reports and textbooks. The material does not attempt to be exhaustive (e.g., without inclusion or exclusion criteria) but rather to highlight key manuscripts that, in the authors’ opinion, contributed to current diagnostic criteria or have addressed their validity. Given the conceptual nature of the topic, this approach was preferred, although its limitations with respect to a systematic review must be acknowledged (e.g., risk of selection bias or lack of systematic synthesis). Based on the review of the available empirical data, a second aim was to propose a reconceptualization of the diagnostic criteria as a rationale for future research aimed at improving the validity of BD. Before beginning, the concept of validity in psychiatry will be briefly introduced, as well as the particularities of its application in BD.

## 2. Validity in Psychiatry

Although there is no unequivocal definition of validity in psychiatry, it can be applied generically to two types of domains: nosological and diagnostic.

### 2.1. Nosological Validity

In the nosological domain, a valid diagnostic category is one that represents a real mental disorder (real entity), that is, it exists in nature by its own right, and not only by convention or human artifice [11]. Knowledge of the etiology (e.g., neurosyphilis) or at least the physiopathogenesis (e.g., Alzheimer’s disease) would be considered the gold standard to demonstrate that a clinical construct is a real entity. In the absence of a known etiology or physiopathogenesis, as in most psychiatric diagnoses, nosological validation has been addressed through two methodologies. The first and most widespread consists of demonstrating that the clinical entity has certain distinctive features such as clinical course, familial aggregation, laboratory data, and response to treatment which are not linked with their definition. This was formalized as external validators by Robins and Guze in 1970 and then reconceptualized by other authors until the latest revision of the DSM-5 [12,13,14,15]. A second strategy is to assess whether there are natural boundaries between related disorders or between a disorder and normality, either by demonstrating zones of rarity or a nonlinear relationship between the symptom profiles and a validating variable such as outcome or heritability [16]. Nevertheless, this second approach has been criticized and is less widely used [17].

### 2.2. Diagnostic Validity

In this domain, the notion of validity overlaps with psychometrics and is usually assessed by how well the criteria for a category portray the clinical entity (i.e., construct validity) [11]. Thus, although related, nosological validity and diagnostic validity would not be redundant concepts (i.e., a given set of diagnostic criteria might appropriately differentiate cases from non-cases of a hypothetical construct -good diagnostic validity-, even though the construct itself might fail to justify itself as a real entity-poor nosological validity-) and both should be addressed.

Despite that, BD-I only requires the presence of manic episodes, in general, BD is manifested by recurrent mood episodes of different polarity. Therefore, the notion of validity should be applied to the different types of mood episodes. That is, it could be that the validity of one mood polarity is not equivalent to that of the other polarity, and as a consequence, affects the joint validity of BD.

## 3. Mania

### 3.1. Nosological Validity

Mania appears to be valid in nosological terms based on the several distinctive features associated with it (e.g., clinical course, familial aggregation, response to treatment, etc.) [18,19,20]. In addition, the main clinical features of mania described historically by expert textbook authors and those included in modern diagnostic criteria are relatively consistent [21]. However, the operationalization of the diagnostic criteria in the RDC and DSM-III introduced certain arbitrariness that could affect its diagnostic validity.

### 3.2. Diagnostic Validity

A first problem refers to the primacy of the mood state. Since the publication of manic-depressive insanity by Kraepelin [22], other expert textbooks authors [21], until some of the first attempts to formalize the diagnosis [23,24], mania was described by three fundamental clinical features: (i) elevated mood, (ii) pressured speech, and (iii) hyperactivity. It is noteworthy that during those years there was no primacy of any clinical feature over another, which made it possible to diagnose mania without any elevation of mood [24]. This historical perspective changed after the publication of the RDC and DSM-III, in which elevated, expansive, or irritable mood was prioritized (Criterion A) over other manic symptoms and signs (Criterion B), coinciding with the inclusion of BD within the ‘mood disorders’ section of the DSM-III. There was no empirical evidence before or after that decision to support the primacy of mood abnormalities over other signs and symptoms of mania. Based on subsequent studies, this was partially reversed in the DSM-5 (published in 2013), in which persistent increased activity or energy was also included as part of Criterion A for the diagnosis of mania; for a review see [25]. However, other studies summarized in a recent review seem to indicate that even some additional clinical features of mania, such as increased speech or flight of ideas, would be at least as relevant as mood abnormalities or increased activity or energy [26]. That review proposed three core features in addition to mood abnormality for diagnosing mania: increased activity, speech, and thought (Table 1).

A second issue related with diagnostic validity is the tonality of mood change in mania. Although traditionally described as elevated or euphoric, dysphoric mood in mania has also been reported from classical texts to recent studies, although not properly included in current diagnostic manuals. For example, depressive mania (i.e., manic symptoms with depressive mood) was described as one of Kraepelin and Weygandt’s mixed states [27] and could not be included as any of the diagnoses available in modern nosologies (neither as mania nor as major depressive episode in RDC or DSM-III or successive editions, nor as mixed episode in DSM-IV, nor as manic or depressive episode with mixed features in DSM-5). Even more, also in his descriptions of pure mania, Kraepelin reported mood changes from euphoria to “anxious, despairing thoughts of death” [22]. This is consistent with accurate and more contemporary descriptions of stages of mania, showing that more than 50% of cases have depressive mood and almost all irritability/mood lability throughout the episode [28,29]. Also, based on a multivariate analysis combining clinical assessment and self-reports in the French EPIMAN study, Akiskal and colleagues proposed a redefinition of mania, in which four mood alterations were specified: elation, depression, anxiety, and irritability [30]. Another recent review on the structure of mania found that elevated mood, irritability, and depression-anxiety were distributed across different dimensions, suggesting that mood in mania might depend on an orthogonal combination of these factors [26]. Finally, a series of interesting studies using different clinical assessment instruments and an emotional induction protocol showed that manic patients have an increase in all emotions [31,32,33,34]. Taken together, the empirical evidence seems to support that the fundamental mood experience in mania is increased mood reactivity to internal or external stimuli (more than just elevated mood or irritability), which can manifest heterogeneously depending on the co-occurrence of different euphoric–dysphoric states of varying intensity (both between patients and within the same patient throughout the manic episode).

## 4. Hypomania

### 4.1. Nosological Validity

Kraepelin used the term hypomania in his description of manic-depressive insanity to denote a clinical form of manic state less pronounced without psychotic or confusional features [22]. Hypomania was redefined by Dunner and colleagues in the 1970s, when they identified in a sample of patients hospitalized for depression a subgroup that clearly had mild elevated mood episodes lasting more than 2–3 days (usually not requiring treatment), but not manic episodes [35,36]. A series of subsequent studies demonstrated that patients with hypomania and depression were more closely related in terms of external validators to BD than to unipolar depression, and as a consequence, bipolar-II disorder (BD-II) achieved formal status in DSM-IV (published in 1994); for a review see [37,38]. Thus, hypomania went from being a clinical form of mania to a different type of mood episode. However, further studies are necessary to nosologically validate whether hypomania is distinct from mania or a milder manifestation of mania [39,40]. In a series of studies of clinically diagnosed BD patients who retrospectively reported their experience during elevated mood episodes, Parker and colleagues found that the only distinguishing features of mania and hypomania were the presence of psychotic symptoms [41,42,43]. Other authors focused on differences in external validators between BD-I and BD-II rather than between manic and hypomanic episodes by themselves, although the research was also sparse and inconclusive. In fact, as Dunner himself acknowledges in a more recent editorial, “differences supporting the separation of bipolar II as a distinct group have not been replicated”, largely due to lack of studies [44]. The few data consistently reported across studies, such as the higher frequency of depressive recurrences or suicidal attempts in BD-II compared with BD-I [37,38] might be better explained in terms of different predominant polarity (i.e., higher prevalence of depressive predominant polarity in BD-II) than of different disorders.

### 4.2. Diagnostic Validity

The operationalization of hypomania in the DSM-IV and successive editions required the same clinical items as mania but, by definition, they are not severe enough to cause marked functional impairment or hospitalization and there are no psychotic symptoms. These criteria have been criticized as vague, since the concept of marked impairment is subjective and difficult to operationalize, and hospitalization depends on multiple variables beyond the clinical severity (e.g., comorbid disorders, poor family support, access to the health system, etc.) [39,45].

Inconsistencies and lack of diagnostic validity were also made regarding the requirement of the 4 days minimal duration of the episode of hypomania. For example, a series of more recent investigations showed that people with hypomania lasting 2–3 days have as many impairments and family history of BD as those with 4 or more days [46,47].

Finally, the same considerations made regarding the diagnostic validity of mania (i.e., primacy of the mood state and tonality of mood change) could be made with respect to hypomania, although there is much less research on the latter

## 5. Bipolar Depression

### 5.1. Nosological Validity

In contrast to mania, there is less overall consistency between the description of depression by authors of classical texts and the clinical features of current diagnostic criteria [48]. Furthermore, the clinical concept of bipolar depression has undergone additional modifications with modern operationalized diagnosis. In fact, in Kraepelin’s descriptions of manic-depressive insanity [22], the first editions of the DSM (1952), the pioneering studies showing the mood stabilizing properties of lithium [49,50], and the research of Leonhard and Perris that led to the separation of BD and unipolar depression [18,19], bipolar depression was conceptualized as melancholic/psychotic and recurrent. However, from the RDC and DSM-III, any type of depressive episode (melancholic or non-melancholic, with psychosis or not, recurrent or not) was considered part of the BD if it occurred in a subject with a history of mania (extended to threshold hypomania in DSM-IV and subthreshold hypomania in DSM-5). This change implicitly assumed that there was no difference between these subtypes of depressive episodes in terms of external validators such as clinical course, familial aggregation, or response to treatment, among others [10]. Despite the lack of empirical evidence to support this assumption, that broad definition of bipolar depression has dominated clinical practice and research for the past four decades. A series of recent small exploratory studies reported that subjects with BD with current or past melancholic depressive episodes showed substantial differences in a number of diagnostic validators relative to those with non-melancholic ones: similar prevalence in men and women (in contrast to higher prevalence in women of non-melancholic); more family history of BD; more severe episodes with a higher prevalence of psychosis, executive-attentional dysfunction and soft neurological signs; a phasic course and better inter-episode functional recovery; and a lower history of suicide attempts [51,52,53,54]. Likewise, some additional analyses showed that melancholic features in BD depression were predictors of good response to pharmacotherapy [55,56]. These findings tend to question the nosological validity of the current broad concept of bipolar depression, suggesting the need for further research to clarify this relevant issue. It is interesting to note that many of the external validators of melancholia (i.e., melancholic depressive episodes) are more similar to those of mania than to those of non-melancholic depressive episodes (Table 2) [51,54,57,58,59,60].

### 5.2. Diagnostic Validity

It is important to highlight that some of the differences in the external validators between melancholic and non-melancholic bipolar depressive episodes mentioned above were found when diagnostic tools other than the DSM-5 specifier (e.g., Sydney Melancholia Prototype Index) were utilized [53,54]. This is consistent with previous criticism of the DSM melancholic features specifier for its overlap with the diagnostic criteria major depressive episode suggesting the poor diagnostic validity of this tool [57,61]. In fact, if the DSM specifier does not allow for an accurate diagnostic distinction of melancholic and non-melancholic depressive episodes (e.g., false positives for melancholia due to overlap with criteria for major depressive episode), then it is more difficult to be able to show differences in external validators between these subtypes in any research. A recent review also showed discrepancies between the clinical items of the DSM-5 specifier and those derived from empirical data for the identification of melancholia [62]. The core clinical features of melancholia that emerged from this review are the opposite of those of mania, suggesting again (i.e., as in mania) that core features such as abnormalities in motor activity, thought and speech are as relevant as the mood change (Table 1). Overall, the diagnostic validity of the current concept of melancholic features and major depressive episode should also be the focus of future studies in BD.

## 6. Discussion

Based on the review of the literature, the concept of mania appears to be a valid construct in nosological terms, as shown by its consistent description over time, and the multiple distinctive validation features associated with it (e.g., clinical course, familial aggregation, response to treatment, etc.). However, some issues regarding its validity in the diagnostic domain warrant further investigation: (i) increased activity, speech, and thought would be as relevant as mood abnormality for the diagnosis of mania; (ii) the increase in mood reactivity rather than just euphoria/irritability would be the fundamental mood change in mania (Table 1). If future research confirms the criteria in Table 1, some clinical pictures conceptualized as mixed or agitated depression [63] could be better diagnosed—and treated—as manic episodes.

In contrast to the consistent evidence regarding the utility of lowering the manic syndrome threshold for diagnosing BD (i.e., duration of mood symptoms for 2–3 days), there are scant and controversial empirical data on the nosological validity of separating hypomania from mania as different episodes (and consequently of separating BD-I and BD-II as distinct entities). Future research should demonstrate the nosological validity of hypomania as an episode distinct from mania and eventually improve its diagnostic validity. Alternatively, one could advocate a single concept of a continuum of mania (lasting 2–3 or more days) manifesting from mild episodes without impaired psychosocial functioning to severe episodes (with or without psychosis) and significant impairment in psychosocial functioning [39,40].

The greatest limitations in terms of nosological and diagnostic validity lie in the current concept of bipolar depression. This concept emerged in the RDC and DSM-III and implicitly assumes that all types of depressive episodes (melancholic or non-melancholic, with or without psychosis, recurrent or not) are equivalent in terms of external validators and, consequently, that if they occur in a subject with a history of (hypo)mania, they are necessarily part of BD. However, there is no empirical evidence available to support this assumption. On the contrary, the few studies that have focused on this topic show differences in external validators between melancholic and non-melancholic episodes (Table 2), questioning the nosological validity of the current concept of bipolar depression [51,52,53,54,55,56]. Therefore, future studies should be conducted in order to improve the nosological validity of bipolar depression considering the possibility that not all types of episodes are equivalent or necessarily constitutive of the illness.

It could be hypothesized that melancholia is a valid clinical entity in nosological terms and, in subjects with a history of (hypo)mania, is constitutive of the BD. This is supported by its historical tradition (e.g., included as part of “manic-depressive insanity”) and the similarity with mania in terms of the core clinical features (although in opposite directions-i.e., increase vs. decrease-) and external validators (Table 2). Psychotic symptoms may appear in severe melancholic episodes, as frequently occurs in severe mania. It is important to note, however, that there is consistent evidence that the DSM’s specifier of melancholic features may have flaws in its diagnostic validity, so other definitions of melancholia should be considered for further research.

On the other hand, non-melancholic/non-psychotic depressive episodes (hereinafter called clinical depression) are a heterogeneous group of clinical pictures, probably of a dimensional nature rather than categorical, that might be defined by diagnostic criteria similar to those of a major depressive episode in the DSM-5 (but without core features of melancholia, Table 1). It could be hypothesized that a person with BD might suffer from (episode/s of) clinical depression without it being necessarily part of their illness, as occurs—with a prevalence of between 20–45—in other psychiatric conditions such as schizophrenia, anxiety disorders, substance abuse disorder, or personality disorders [64,65,66]. This hypothesis is compatible with some theoretical models proposing that mania and (DSM-defined) major depressive episode is too broad and heterogeneous [67,68], and with some familial studies showing independence in the inheritance of mania and depression in BD using current diagnostic criteria [69,70,71]. The same can be extended to subthreshold depressive symptomatology, which is usually interpreted as a clinical manifestation of BD (i.e., part of the illness); for a review see [72], although it also might have other origins. In fact, there are multiple conditions that can trigger threshold or subthreshold depressive symptoms in any subject, and all of them are overrepresented in patients with BD: chronic stress associated with life adversities, side effects of mediations, alcohol or substance misuse, and medical or psychiatric comorbidities (e.g., hypothyroidism or anxiety disorders) illnesses. If indeed clinical depression (or some subthreshold depressive symptoms) was not part of BD, it might not respond to the usual bipolar therapeutic algorithms. Consequently, the current broad concept of bipolar depression could lead to overmedicating some patients, or depriving them of other more effective therapeutic strategies. Furthermore, the identification of these clinical depressions in subjects with predictors of bipolarity or subthreshold hypomania [46,73] could lead to the misdiagnosis of BD, contributing to the risk of overdiagnosis reported in the literature [74,75,76,77,78]. Finally, the real prevalence of unipolar mania might be substantially higher than that estimated with current broad concept of bipolar depression. Therefore, the nosological and diagnostic validity of these clinical depressions in the context of BD should be the focus of future research.

**Figure 1:**
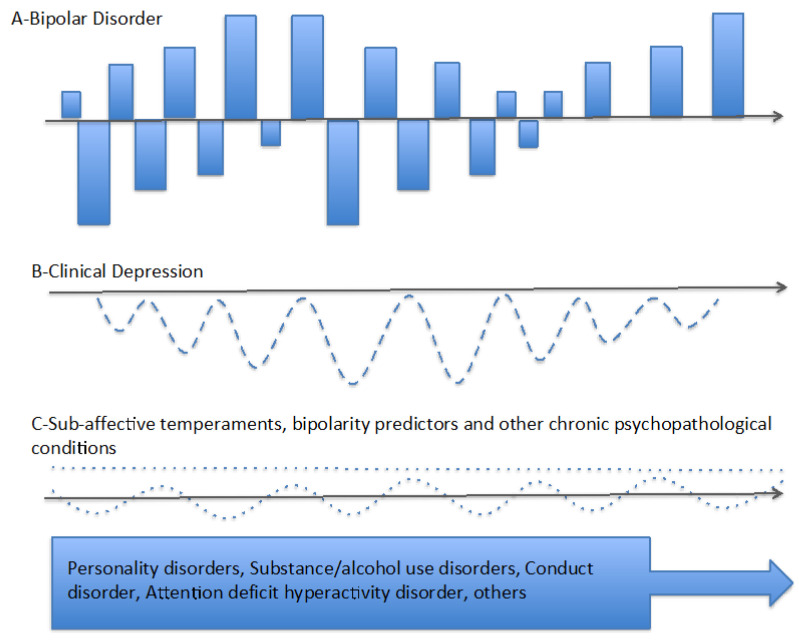
Proposed tentative model for bipolar disorder (BD). (**A**) Diagnosis of bipolar disorder (BD) requires the occurrence of episodes of mania with or without melancholic depression. The severity of episodes of mania and melancholia might range from mild to severe with or without psychosis. (**B**) Mild to severe episodes of clinical depression might occur in patients with BD without being part of their illness, as occurs in other psychiatric conditions such as schizophrenia, anxiety disorders, substance abuse, or personality disorders. (**C**) Affective temperaments (i.e., hyperthymic or cyclothymic temperament in dotted line) and other predictors of bipolarity, as well as certain chronic psychopathological conditions might occur with some frequency in patients with BD. Identifying predictors of bipolarity in patients with unipolar melancholia might contribute to the early diagnosis of BD. However, the presence of these same predictors or other chronic psychopathological conditions in subjects with clinical depression (but without mania or melancholia) could lead to a misdiagnosis of BD.

Taken together, the broad current definition of bipolar depression could include true episodes of the illness (e.g., melancholic/psychotic depression), others that could be better conceptualized as manic episodes (e.g., some mixed or agitated depression), or others that are not even part of BD (e.g., some clinical depression in the context of life adversities). This illustrates how the lack of nosological validity could impede the advancement of our knowledge about the pathophysiological substrate of BD or the availability of effective personalized treatments. Based on the empirical data reviewed, a tentative reconceptualization of the diagnosis of BD is proposed for further research: it requires the recognition of manic episodes with or without melancholia (according to Table 1). Both manic and melancholic episodes can range from mild to severe with or without psychotic symptoms (Figure 1A). On the other hand, mild to severe episodes of clinical depressions might occur in patients with BD without being part of their illness (Figure 1B). Finally, there would be other conditions with high comorbidity with both BD and clinical depression that include from normal sub-affective temperaments to personality disorders, substance use disorders, ADHD, or conduct disorders (Figure 1C). This model suggests that BD (i.e., mania and melancholia), clinical depression, and other psychopathological conditions associated with mood swings, even with the possibility of overlap in some patients, would have different pathophysiological substrates and require tailored therapeutic approaches. If this were the case, the usual bipolar therapeutic algorithms (e.g., lithium) would be useful for the treatment and prophylaxis of mania and melancholia, but not necessarily for clinical depression or other conditions associated with mood swings. This perspective diverges from dimensional models that propose that all types of mood swings might depend on a common pathophysiological process or require the same treatments. Of course, the proposed tentative model does not attempt to solve the problem of the validity of the BD, but to generate hypothesis for future research aimed at improving it both in nosological and diagnostic terms. Importantly, future research could benefit from considering certain methodological aspects: (i) larger sample sizes; (ii) diagnostic tools that include but are not limited to DSM criteria; (iii) not combining clinical conditions (e.g., melancholic and non-melancholic depressive episodes) before their equivalence in terms of nosological validity has been demonstrated; and (iv) not promoting new categories (e.g., bipolar spectrum depression) before demonstrating the diagnostic and nosological validity of a higher hierarchy category (e.g., bipolar depression).

## 7. Conclusions and Future Directions

The validity of the current diagnosis of BD has been a relatively neglected topic and warrants further research. Mania and melancholia appear to be valid constructs in nosological terms, although their diagnostic validity could be improved. The nosological and diagnostic validity of hypomania and non-melancholic/non-psychotic depressive episodes is limited. Any approach using technology (from biomarkers and genetics to machine learning and artificial intelligence) aimed at improving the diagnosis, pathophysiological or therapeutic understanding of BD will be more fruitful if applied to a nosologically valid definition of it.

## Figures and Tables

**Table 1:** Proposed revised diagnostic criteria for mania, melancholia, and clinical depression.

Mania	Melancholia	Clinical Depression
A distinct period of abnormality lasting at least 3 days (or any duration if hospitalization is necessary) in which five or more of the following criteria are met: A–Increased motor activity, speech, and thought (at least one). -Increase in goal directed activity or psychomotor agitation -More talkative than usual or pressure to keep talking -Flight of ideas B–Increased mood reactivity (at least one): -Elevated or expansive mood -Irritability -Mood lability -Depressive mood C–Other manic features –Expansiveness, over-confidence, or grandiosity (overvalued idea or delusion) –Decreased need for sleep –Distractibility –Excessive involvement in activities that have a high potential for painful consequences –Additional features (lack of insight/disruptive-aggressive behavior).	A distinct period of abnormality lasting at least 2 weeks in which five or more of the following criteria are met: at least one of the features must be from section A or B A–Retarded motor activity, speech, and thought (at least one). –Motor retardation: slowness of movement, facial and body immobility –Retardation speech and thought: delay in responding verbally, slowing of speech rate, impaired spontaneity of talk, or short length of verbal responses B–Decreased mood reactivity: decreased ability to improve or worsen mood in response to the environment C–Other melancholic features -Pathological guilt/self-reproach (overvalued idea or delusion) -Distinct quality of depressive mood -Loss of pleasure/anhedonia -Middle or late insomnia -Significant loss of appetite or weight -Diurnal variation (worse in morning)	A distinct period of abnormality lasting at least 2 weeks in which five or more of the following criteria are met: at least one of the symptoms is either (1) depressed mood or (2) loss of interest or pleasure. 1- Depressed mood 2- Markedly diminished interest or pleasure 3- Significant weight loss when not dieting or weight gain 4- Insomnia or hypersomnia nearly every day 5- Fatigue or loss of energy nearly every day 6- Reluctance for usual activities 7- Diminished ability to think or concentrate, or indecisiveness 8- Feelings of worthlessness, guilt, hopelessness, or other depressive cognitions 9- Recurrent thoughts of death (not just fear of dying), recurrent suicidal ideation without a specific plan, or a suicide attempt or a specific plan for committing suicide Diagnostic criteria for melancholia are not met

In any of the three diagnoses, two additional criteria are met: 1—The symptoms causes an unequivocal change—corroborated by third parties—in the normal psychosocial functioning of the individual. 2—The episode is not mainly attributable to the physiological effects of a substance or another psychiatric or medical condition.

**Table 2:** Comparison of core features and external validators of mania, melancholia and clinical depression.

Mania	Melancholia	Clinical Depression
1-Core clinical features -Increased mood reactivity -Increased motor activity -Increased speech and thought	1-Core clinical features -Decreased mood reactivity -Motor retardation -Retardation of speech and thought	1-Core clinical features -Depressed mood -Loss of interest or pleasure
2-Frequent mood-congruent overvalued (or delusional) ideas	2-Frequent mood-congruent overvalued (or delusional) ideas	2-Infrequent delusions and hallucinations
3-Familial aggregation in bipolar disorder	3-Familial aggregation in bipolar disorder	3-Less family history of bipolar disorder
4-Usually adequate premorbid adjustment	4-Usually adequate premorbid adjustment	4-More frequent history of personality disturbances
5-Phasic rather than chronic clinical course	5-Phasic rather than chronic clinical course	5-More chronic course, frequent subclinical symptoms
6-Male:female relationship close to unity	6-Male:female relationship close to unity	6-More frequent in female than male
7-Positive response to lithium	7-Positive response to lithium	7-No response to lithium

## Data Availability

No new data were created or analyzed in this study. Data sharing is not applicable to this article.
